# Addressing Unmet Needs in Heart Failure with Preserved Ejection Fraction: Multi-Omics Approaches to Therapeutic Discovery

**DOI:** 10.3390/ijms27020673

**Published:** 2026-01-09

**Authors:** Taemin Kim, Michael Sheen, Daniel Ryan, Jacob Joseph

**Affiliations:** 1 Warren Alpert Medical School, Brown University, Providence, RI 02903, USA; tae_min_kim@brown.edu (T.K.); michael_sheen@brown.edu (M.S.);; 2 Cardiology Section, VA Providence Healthcare System, Providence, RI 02908, USA

**Keywords:** heart failure with preserved ejection fraction, heterogeneity, endophenotypes, multi-omics, systems biology, precision cardiology, biomarkers, clinical trial design

## Abstract

Heart failure with preserved ejection fraction (HFpEF) accounts for about half of heart failure cases and is linked to aging, obesity, diabetes, and multimorbidity, yet disease-modifying therapies remain limited. A major barrier is heterogeneity: HFpEF comprises overlapping inflammatory, fibrotic, cardiometabolic, and hemodynamic/vascular endophenotypes embedded within systemic cardiorenal and cardiohepatic cross-talk, which conventional metrics such as left ventricular ejection fraction (LVEF), natriuretic peptides (NPs), and standard imaging capture incompletely. In this narrative review, we synthesize clinical, mechanistic, and trial data to describe HFpEF endophenotypes and their multi-organ interactions; critically appraise why traditional diagnostic and enrollment strategies contributed to neutral outcomes in landmark trials; and survey emerging cardiovascular multi-omics studies. We then outline an integrative systems-biology framework that applies (i) within-layer analyses and cross-layer integration, (ii) network-based driver nomination and biomarker discovery, and (iii) target nomination to link molecular programs with circulating markers and candidate therapies. Finally, we discuss practical challenges in implementing multi-omics HFpEF research and highlight future directions such as artificial intelligence (AI)-enabled multi-omics integration, cross-organ profiling, and biomarker-guided, endotype-enriched platform trials. Collectively, these advances position HFpEF as a proving ground for precision cardiology, in which therapies are matched to molecularly defined disease programs rather than ejection-fraction cutoffs alone.

## 1. Introduction

Heart failure (HF) is a complex syndrome in which the heart struggles to maintain sufficient cardiac output to meet the body’s metabolic demands, leading to symptoms such as dyspnea, fatigue, and fluid retention [1]. HF is divided into three major subtypes based on left ventricular ejection fraction (LVEF): heart failure with reduced ejection fraction (HFrEF; LVEF < 40%), heart failure with mildly reduced ejection fraction (HFmrEF; 40–49%), and heart failure with preserved ejection fraction (HFpEF; ≥50%) [2]. While HFrEF primarily reflects impaired contractility, HFpEF arises from abnormalities in relaxation and increased ventricular stiffness despite mostly preserved contractile function [3,4]. Among these phenotypes, HFpEF has become increasingly prevalent—particularly in older adults and individuals with metabolic comorbidities such as obesity and diabetes—and now represents one of the most pressing unmet needs in cardiovascular medicine [5,6].

In the United States, an estimated 6.7 million adults are living with HF, a number projected to exceed 8.5 million by 2030 [7]. HFpEF currently accounts for approximately half of all heart failure (HF) cases [8]. Patients with HFpEF experience substantial morbidity, frequent hospitalizations, and high long-term mortality, approaching 50–75% at five years—rates comparable to those observed in HFrEF [7,8]. The condition also poses a major economic burden, with U.S. healthcare costs for HF estimated at $31 billion in 2012 and projected to rise to $70 billion by 2030, primarily due to hospitalization expenses [9]. Despite this impact, effective therapies for HFpEF remain limited—standing in stark contrast to HFrEF, for which multiple pharmacologic classes, including angiotensin-converting enzyme (ACE) inhibitors/angiotensin receptor blockers (ARBs), angiotensin receptor–neprilysin inhibitors (ARNIs), mineralocorticoid receptor antagonists (MRAs), and beta-blockers, have demonstrated consistent survival benefits [10,11,12].

HFpEF presents considerable clinical and pathophysiological challenges, primarily stemming from its heterogeneity. It is increasingly recognized not as a single disease but as an umbrella syndrome encompassing diverse endophenotypes characterized by fibrosis, inflammation, cardiometabolic dysfunction, atrial fibrillation, pulmonary hypertension, anemia, and obesity [10,13,14]. Further complicating diagnosis and patient stratification are inconsistencies in ejection fraction cutoff thresholds and diagnostic criteria, which lead to variable trial populations [15].

Addressing these challenges necessitates a paradigm shift towards precision stratification [16]. This approach focuses on identifying distinct endophenotypes, such as those driven by fibrosis and inflammation, and understanding their association with comorbid clusters like atrial fibrillation, pulmonary hypertension, obesity, and anemia [17]. Traditional biomarkers and imaging, while useful, often fall short in capturing this intricate heterogeneity at a granular level [18].

In this context, the promise of multi-omics approaches and advanced translational models offers a transformative path forward [19]. The integration of multi-omics data has already revolutionized oncology, providing a powerful precedent [20]. A similar approach in HFpEF may unlock disease-modifying strategies where traditional methods have failed. Multi-omics, including genomics, transcriptomics, proteomics, metabolomics, and single-cell/spatial omics, provides unprecedented depth in characterizing disease mechanisms [12]. Furthermore, systems-level insights—achieved by integrating multiple omics layers and studying HFpEF as an interconnected biological system—move beyond isolated technologies to provide a more comprehensive understanding of disease mechanisms, with the promise of identifying key drivers and pathways in disease progression [21,22]. Complementing these are organoid and ex vivo systems, extending beyond cardiac models to include kidney and liver tissues, to reflect the systemic nature of HFpEF [23,24,25,26]. Together, these approaches hold promise for biomarker-driven enrichment strategies, surrogate endpoint development, and targeted drug discovery in HFpEF [12,27,28].

Ultimately, HFpEF is not merely a growing medical concern but serves as a critical “stress test” for precision medicine in cardiology. With its rising prevalence in aging societies, HFpEF represents a looming public health crisis [29]. Success in applying multi-omics and precision strategies to unravel the heterogeneity and target distinct disease mechanisms in HFpEF could validate similar approaches across other complex cardiovascular diseases, demonstrating the transformative potential of precision cardiology as a whole [19].

## 2. The Challenge of Heterogeneity in HFpEF

Despite shared clinical features (impaired relaxation, elevated filling pressures, and exercise intolerance), patients reach HFpEF through distinct biological routes (inflammatory, metabolic, fibrotic, and vascular) [10,30,31].

### 2.1. Defining Endophenotypes in HFpEF

Recent work using clinical clustering, imaging, and omics data supports the idea that HFpEF is composed of several endophenotypes—subgroups of patients who share similar pathobiologic mechanisms despite having the same clinical diagnosis [14,17,32]. Across classification schemes, four major categories commonly emerge: inflammatory, fibrotic, cardiometabolic, and hemodynamic/vascular phenotypes [16,32] (Table 1).

#### 2.1.1. The Inflammatory Endophenotype

Inflammation plays a central role in many cases of HFpEF [33,34]. Patients with obesity, diabetes, hypertension, and CKD often exhibit elevated inflammatory cytokines (e.g., interleukin-6 (IL-6), tumor necrosis factor alpha (TNF-α), C-reactive protein (CRP)), endothelial activation, impaired nitric oxide signaling, and oxidative stress that promote ventricular stiffening [35,36,37,38,39].

Mechanistic support comes from myocardial studies showing increased cardiomyocyte passive tension in HFpEF, implicating reduced phosphorylation of sarcomeric proteins such as titin [40]. This aligns with the comorbidity-driven model in which systemic inflammation blunts nitric oxide (NO)- cyclic guanosine monophosphate (cGMP)-protein kinase G (PKG) signaling and titin phosphorylation, promoting cardiomyocyte stiffness [41].

#### 2.1.2. The Fibrotic Endophenotype

A second major pathway involves fibrosis, characterized by the excessive accumulation of extracellular-matrix proteins such as collagen and fibronectin in the heart [42,43], leading to increased ventricular stiffness and impaired relaxation [10,44]. Mechanistically, transforming growth factor-β signaling alters myocardial matrix composition, while reduced titin phosphorylation increases cardiomyocyte stiffness independent of fibrosis burden [12,45,46,47,48].

Human biopsy studies from Javier Díez and colleagues have shown that myocardial collagen volume fraction correlates closely with circulating collagen propeptides (PICP, PIIINP), establishing collagen turnover as a measurable plasma biomarker of cardiac fibrosis [49]. Proteomic analyses have detected increased levels of matricellular proteins like galectin-3 associated with fibrotic remodeling and inflammation [50,51,52].

#### 2.1.3. The Cardiometabolic Endophenotype

The cardiometabolic or obese phenotype links metabolic dysfunction to cardiac inflammation and energy imbalance [53,54]. Obesity, insulin resistance, and dyslipidemia promote lipotoxicity, mitochondrial dysfunction, inflammatory responses, and microvascular impairment within the myocardium [45,55]. Multi-omics studies have demonstrated altered pathways, including reduced fatty-acid oxidation, and changes in branched-chain amino-acid (BCAA) metabolism and acylcarnitine profiles in these patients [35,36].

An important diagnostic feature is that obese patients often have disproportionately low NP levels, which can lead to underdiagnosis when standard B-type natriuretic peptide (BNP) or N-terminal pro–B-type natriuretic peptide (NT-proBNP) cut-offs are applied [37,38].

#### 2.1.4. The Hemodynamic/Vascular Endophenotype

Another cluster of HFpEF patients is defined by impaired ventricular–vascular coupling and elevated filling pressures, often accompanied by pulmonary hypertension, right-ventricular dysfunction, and increased arterial stiffness [10,39,56]. Symptoms arise primarily from limited ventricular and vascular reserve rather than overt inflammation or fibrosis [56].

This phenotype is enriched among older, hypertensive patients—particularly post-menopausal women—and may preferentially respond to therapies targeting vascular function and ventricular–vascular interaction rather than myocardial contractility alone [10,57].

### 2.2. Shared Comorbidities and Systemic Cross-Talk

HFpEF is not confined to the heart; it reflects multi-organ dysfunction driven by systemic comorbidities [6,26,58]. The cardiorenal axis exemplifies this interaction, whereby elevated venous pressures and chronic kidney disease impair renal function, activate neurohormonal pathways, and accelerate adverse cardiac remodeling [59,60]. Similarly, the cardiohepatic axis contributes to disease progression through hepatic congestion-induced inflammation, fibrosis, and bile acid dysregulation linked to systemic metabolic disturbance [61,62,63]. Together, these pathways highlight the role of multi-organ immune and metabolic crosstalk in shaping HFpEF progression [46].

### 2.3. Why Heterogeneity Explains Trial Failures

The presence of multiple overlapping endophenotypes helps explain why many HFpEF clinical trials have produced neutral or modest results [18,64]. Historically, enrollment has relied on ejection-fraction thresholds and symptoms rather than underlying biological drivers [32,65]. As a result, therapies targeting specific mechanisms were diluted across a mixed population in which only a subset of patients would be expected to benefit [17]. Recognizing this complexity is the first step toward designing biologically enriched trials that align treatment mechanisms with patient endophenotypes [16,18].

### 2.4. Translational Implications of Heterogeneity

Appreciating the biological diversity within HFpEF does more than explain past trial outcomes—it provides a framework for refining diagnosis and treatment [30,32]. Identifying mechanistically defined patient groups enables tailoring of biomarker panels, imaging strategies, and therapeutic targets to dominant disease pathways [16,47]. Integrative multi-omics and advanced clustering tools are increasingly uncovering reproducible molecular signatures corresponding to these endophenotypes [17,48]. Translating these insights into clinical practice will require improved trial design, standardized diagnostic criteria, and practical integration of molecular profiling into patient care, representing a critical bridge between HFpEF heterogeneity and precision medicine [18,49,65,66].

## 3. Limitations of Traditional Diagnostics and Therapeutics

Despite significant advances in understanding HFpEF biology, clinical diagnosis and management remain reliant on traditional tools that only partially reflect the syndrome’s systemic and heterogeneous nature [5]. LVEF, NPs, and standard imaging parameters—cornerstones of heart-failure evaluation—provide only partial insights into the HFpEF pathophysiology [10,30]. Because these tools were optimized for systolic HF, they often fail to resolve HFpEF mechanisms driven by diastolic dysfunction, inflammation, and multiorgan involvement [11,18,67]. Understanding the limitations of these conventional tools clarifies why mechanistic insights have not yet translated into consistent clinical efficacy.

### 3.1. Why Ejection Fraction, Natriuretic Peptides, and Imaging Fall Short

These limitations are most apparent when evaluating how conventional indices perform in HFpEF.

#### 3.1.1. Ejection Fraction

Ejection fraction is a global measure of systolic pump function that inadequately captures HFpEF pathophysiology, where symptoms arise from impaired relaxation and filling despite preserved contractility [12,68]. Patients with identical LVEF can have markedly different diastolic stiffness, relaxation, and filling pressures [10,69].

Variability in EF measurement methods and threshold definitions, along with overlap between HFpEF and HFmrEF (EF 40–49%), has blurred EF-based categories and contributed to heterogeneous trial populations that obscure mechanism-specific treatment effects [5,18,67,70].

#### 3.1.2. Natriuretic Peptides

BNP and NT-proBNP are established heart-failure biomarkers but have limited utility in HFpEF [38]. NPs reflect wall stress but are strongly modified by obesity, renal function, age, sex, and atrial rhythm, reducing diagnostic specificity in HFpEF [38,71,72,73].

As a result, patients with true HFpEF—especially those with obesity or early disease—may have “normal” BNP values and be underdiagnosed or excluded from clinical trials [38,74]. NP thresholds can materially influence trial eligibility and enrich for biologically distinct subgroups, contributing to variable treatment effects [75,76,77].

#### 3.1.3. Imaging Modalities

Echocardiography remains the primary imaging tool for HFpEF evaluation, but commonly used parameters overlap substantially with changes seen in normal aging, hypertension, and atrial fibrillation, limiting diagnostic specificity [6,10,78].

Cardiac magnetic resonance imaging enables tissue-level assessment of fibrosis (e.g., T1 mapping and extracellular volume fraction) but is limited by cost, availability, and expertise requirements, while invasive hemodynamic testing—though definitive—remains impractical for routine use [67,79,80,81].

Collectively, limitations of LVEF, NPs, and imaging highlight the gap between observable phenotype and molecular mechanism, contributing to inconsistent patient selection and underrecognition of HFpEF’s biological diversity [69,82].

### 3.2. Lessons from Major Clinical Trials

The consequences of these diagnostic shortcomings are evident in the mixed results of two decades of HFpEF therapeutic trials. Nearly all major studies enrolled patients based on EF thresholds and symptoms rather than mechanistic profiles, resulting in heterogeneous cohorts and diluted treatment effects [18,65] (Table 2).

#### 3.2.1. TOPCAT

The Treatment of Preserved Cardiac Function Heart Failure with an Aldosterone Antagonist trial evaluated spironolactone in patients with EF ≥ 45% and was neutral overall, despite reduced hospitalizations in the Americas [11,75,83]. Marked regional differences and heterogeneity in enrollment criteria—particularly NP thresholds versus prior hospitalization—yielded biologically distinct subgroups with differential treatment response [11,75,84]. Latent class analyses further support the presence of HFpEF subphenotypes with variable responsiveness to mineralocorticoid receptor antagonism [17].

#### 3.2.2. PARAGON-HF

The Prospective Comparison of ARNI with ARB Global Outcomes in HF with Preserved Ejection Fraction trial evaluated sacubitril–valsartan versus valsartan in patients with EF ≥ 45% and narrowly missed its primary endpoint [61,85]. Subgroup signals in women and in patients with EF 45–57% highlight how modest differences in EF thresholds and sex-linked phenotypic differences (including natriuretic peptide expression) can obscure treatment effects in HFpEF [57,61].

#### 3.2.3. EMPEROR-Preserved

This study tested the sodium–glucose cotransporter-2 inhibitor empagliflozin in patients with EF > 40% and demonstrated a significant reduction in HF hospitalizations without a consistent mortality benefit [62]. Benefits were consistent across EF ranges and diabetes status, consistent with the systemic metabolic, renal, and hemodynamic actions of SGLT2 inhibitors [62,63,86].

#### 3.2.4. DELIVER

The Dapagliflozin Evaluation to Improve the Lives of Patients with Preserved Ejection Fraction Heart Failure trial confirmed that dapagliflozin reduced HF hospitalizations across EF > 40%, including patients with improved EF [87]. Concordance with EMPEROR-Preserved reinforces that therapies targeting systemic metabolic and renal pathways can transcend EF-based classification [63,87,88]. Beyond reductions in heart failure hospitalization, recent adjudicated meta-analyses of randomized trials suggest that empagliflozin and dapagliflozin are associated with a reduced risk of sudden cardiac death across cardio-renal-metabolic populations, including heart failure cohorts, extending the spectrum of cardiovascular benefits attributed to SGLT2 inhibition [89].

### 3.3. The Need for Phenotype-Specific Enrichment Strategies

The neutral or modest outcomes of these landmark trials reveal a consistent theme: diagnostic and enrollment strategies that ignore biological diversity inevitably dilute therapeutic signals [18,32]. Different HFpEF phenotypes are driven by distinct mechanisms and may require different therapeutic strategies [65,90].

Standard endpoints may also miss clinically meaningful benefits within specific HFpEF subgroups (e.g., congestion or exercise tolerance) even in the absence of mortality effects [10,91].

Moving forward, enrichment strategies that incorporate molecular and phenotypic markers—including omics biomarkers and imaging- or metabolism-derived signatures—will be essential for mechanism-aligned trial design [17,32,92,93].

Such approaches would align therapeutic mechanisms with the biological substrate of disease rather than with arbitrary EF thresholds [18]. This need for mechanism-aligned enrichment motivates multi-omics approaches described in the next section [12].

## 4. Multi-Omics in Cardiovascular Research: Principles and Promise

Advances in high-throughput technologies now enable a fully integrated multi-omics characterization of complex diseases, allowing investigators to capture biology across molecular layers [12]. Genomic analyses identify inherited and acquired DNA variants that confer susceptibility or modify therapeutic response, while epigenomic profiling reveals regulatory mechanisms—such as DNA methylation and histone modification—that shape gene expression in response to environmental or metabolic stressors. Transcriptomic approaches provide dynamic measurement of RNA expression programs, often uncovering cell-type-specific responses activated during inflammation, oxidative stress, or mechanical overload. Proteomics extends this framework by quantifying proteins and post-translational modifications that directly mediate cellular function, and metabolomics profiles small-molecule intermediates that reflect real-time physiological states and energetic fluxes. Furthermore, single-cell and spatial omics resolve tissue heterogeneity by mapping how discrete cell populations and their microenvironments contribute to disease remodeling. Together, these complementary modalities establish a systems-level framework that links genotype to phenotype, enabling identification of causal pathways and therapeutic targets that remain invisible to single-platform analyses [12,68].

### 4.1. Lessons from Other Disciplines

The transformative potential of multi-omics has been best illustrated in oncology and nephrology [94,95]. In cancer research, large-scale initiatives such as The Cancer Genome Atlas integrated genomic, transcriptomic, and proteomic data to redefine tumors based on molecular signatures rather than organ of origin [96,97]. This molecular taxonomy directly guided the development of targeted therapies—HER2 amplification leading to trastuzumab in breast cancer [98], EGFR and ALK inhibitors in lung cancer [99], and immune-checkpoint therapies guided by tumor mutational burden [100]. Similarly, in kidney disease, integrated transcriptomic and proteomic analyses of biopsy tissue have identified distinct molecular programs in diabetic nephropathy [101] and delineated molecular subgroups of lupus nephritis, improving diagnostic precision and revealing actionable immune pathways, particularly IFN-γ-inducible chemokine signaling [102]. These precedents demonstrate that multi-omics integration can transform phenotypically defined syndromes into mechanistically stratified diseases with targeted treatment options.

### 4.2. Emerging Omics Insights in HFpEF

Comparable integrative efforts are now underway in cardiovascular research. Early proteomic studies have identified multi-protein circulating signatures enriched for inflammation (e.g., LCN2, U-PAR, IL-1ra, Gal-9) and extracellular-matrix remodeling (e.g., TIMP-1, MMP7, MATN2) that differentiate HFpEF from HFrEF and, in some cohorts, predict HF hospitalization and mortality [103,104]. Metabolomic analyses have identified abnormalities in fatty-acid oxidation pathways and highlighted coordinated variation within BCAA metabolism, consistent with altered myocardial energetics and systemic metabolic stress [35,105]. Transcriptomic and single-cell RNA sequencing (RNA-seq) approaches in human and experimental HF are uncovering discrete cardiomyocyte, fibroblast, endothelial, and immune-cell states, revealing subtype-specific inflammatory, fibrotic, and metabolic programs that distinguish HFpEF from HFrEF [13,106]. Spatially resolved transcriptomic approaches—including multiplex single-molecule fluorescence in situ hybridization (smFISH)—are further defining micro-regional communication between cardiomyocytes and non-myocyte cells, offering unprecedented resolution into tissue remodeling [107].

Although still in their early stages, these omics initiatives lay the groundwork for a molecular taxonomy of HFpEF—one that integrates systemic and cardiac data to delineate mechanistic subtypes, guide biomarker development, and inform precision-guided clinical trials. As analytical pipelines mature and data harmonization improves, multi-omics integration holds the promise to bridge the gap between mechanistic discovery and therapeutic efficacy in HFpEF.

## 5. Integrative Multi-Omics Framework for HFpEF

HFpEF’s heterogeneity necessitates a systems-biology perspective in which molecular layers are analyzed jointly to reveal shared structure across genomic variation, chromatin state, RNA expression, proteins, metabolites, and cell states. This approach typically involves three complementary analytic steps: (i) within-layer modeling to identify disease-associated features and enriched pathways in each omics dataset (e.g., differentially expressed genes, proteins, or metabolites involved in inflammatory or fibrotic signaling); (ii) cross-layer integration to align multiple omics modalities and uncover shared latent factors that represent coordinated molecular programs spanning genes, proteins, and metabolites; (iii) network inference to construct interaction maps that link correlated molecules into modules and highlight hub regulators driving disease processes.

Matured analytic frameworks in cardiovascular multi-omics include matrix-factorization and multi-view methods (e.g., canonical correlation analysis (CCA), Multi-Omics Factor Analysis (MOFA)-style latent factors), co-expression and co-abundance networks (Weighted Gene Co-expression Network Analysis (WGCNA)-like modules linking genes, proteins, and metabolites to phenotypes), and graph-based models embedding multi-omic features onto protein–protein or ligand–receptor interaction networks. Collectively, these approaches delineate endophenotype-specific modules—such as inflammatory, fibrotic, and cardiometabolic signatures—and link them to clinically relevant remodeling and outcome phenotypes in heart failure [13,21,22,108].

### 5.1. An Actionable Pipeline for Integrated Omics in HFpEF

**Figure 1:**
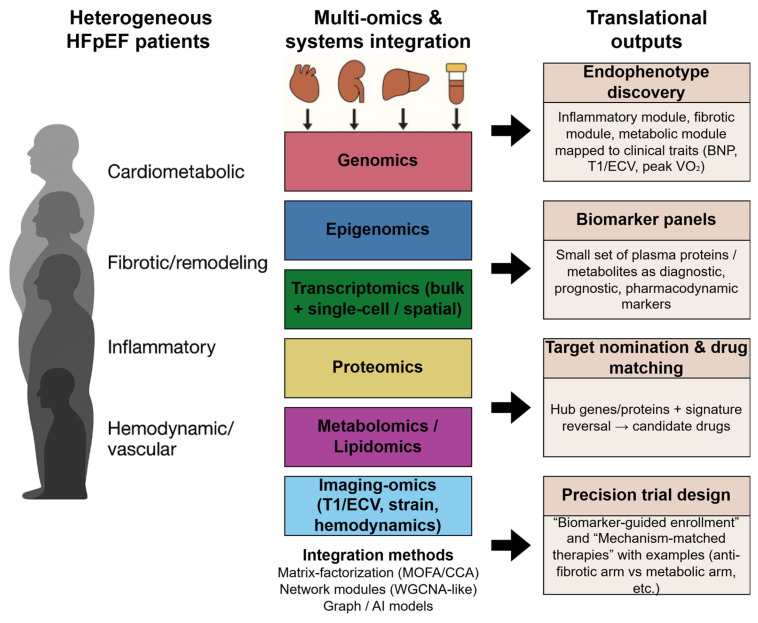
Integrative multi-omics pipeline linking HFpEF heterogeneity to endophenotype discovery, biomarker development, and mechanism-matched therapies. Heterogeneous HFpEF phenotypes (**left**) undergo multi-layer molecular profiling and systems integration (**center**), including genomics, epigenomics, transcriptomics, proteomics, metabolomics, and imaging-omics. Integration methods (e.g., Multi-Omics Factor Analysis (MOFA)/canonical correlation analysis (CCA), network modules, graph/artificial intelligence (AI) approaches) yield translational outputs (**right**) such as endophenotype modules, biomarker panels, drug-target nomination (via signature reversal, i.e., drugs that oppose the HFpEF molecular pattern), and precision trial designs.

With these integrative frameworks in place, the next step is to operationalize them into a pipeline that prioritizes causal drivers, identifies accessible biomarkers, and matches HFpEF molecular signatures with candidate therapies (Figure 1).

#### 5.1.1. Driver Nomination (Causal Prioritization)

Genetic association signals can be linked to molecular regulation by performing expression quantitative trait locus (eQTL) and protein quantitative trait locus (pQTL) colocalization [109] as well as Transcriptome-Wide Association Studies (TWAS) [110]. These analyses identify variants that influence gene or protein abundance [17] and can be cross-referenced with myocardial single-cell or spatial transcriptomic maps to assign cell-type specificity [107]. Complementary perturbation analyses—such as overlaying Clustered Regularly Interspaced Short Palindromic Repeats (CRISPR) or siRNA knock-down signatures, or drug-response transcriptomes—test whether modulating a candidate gene reverses HFpEF-like molecular patterns, providing causal evidence [111].

#### 5.1.2. Biomarker Discovery (Diagnostic, Prognostic, and Pharmacodynamic)

Molecular network analyses group co-regulated genes, proteins, or metabolites into modules—sets of features that rise and fall together because they participate in shared biological programs (e.g., inflammatory, fibrotic, or metabolic signaling). The overall activity of each module can then be summarized by its eigengene or latent factor, a single quantitative score capturing the dominant expression pattern within that module [112,113]. By correlating these module scores with HFpEF traits (e.g., diastolic dysfunction, impaired energetics), investigators can identify which biological programs are most tightly linked to specific clinical endophenotypes. These module-level scores provide compact, biologically interpretable readouts of HFpEF-relevant processes and can be linked to clinical phenotypes.

Circulating proxies—including plasma proteins, eicosanoids, and acylcarnitines—that associate with HFpEF-related echocardiographic and exercise traits (e.g., E/e′, peak VO_2_ (peak oxygen consumption), surrogates of left-atrial pressure) provide accessible biomarker candidates [35,108,114]. Regularized regression models such as lasso [115] and elastic-net [116] enable selection of compact, non-redundant biomarker panels that can be validated across cohorts and HFpEF endotypes (e.g., obese vs. fibrotic). Pharmacodynamic markers—those that change in response to targeted therapy—further support mechanism-matched treatment monitoring.

#### 5.1.3. Target Nomination and Drug Matching

Candidate therapeutic targets are ranked by integrating network centrality—the position of genes or proteins within co-expression or protein–protein interaction networks [117,118,119]—with genetic evidence from eQTL or TWAS analyses [12,120], as well as druggability metrics reflecting ligandability or structural tractability [121]. Signature-matching approaches, based on the Connectivity Map framework, compare HFpEF molecular profiles with drug-induced gene-expression signatures to identify compounds predicted to reverse disease states [122,123]. These computational predictions can inform biomarker-guided clinical trials, for example, a trial in which patients with high fibrosis-module scores are enriched for anti-fibrotic therapies and collagen-related pharmacodynamic markers serve as secondary endpoints [124,125].

Together, this pipeline connects molecular causality, measurable biomarkers, and therapeutic targeting within a unified systems-medicine framework [12].

## 6. Practical Challenges and Solutions in Multi-Omics HFpEF Research

While this framework outlines a coherent path toward mechanism-guided therapies for HFpEF, its practical implementation faces several methodological and logistical challenges.

### 6.1. Cohort Design and Phenotyping

Multi-omics studies in HFpEF often rely on small, heterogeneous cohorts with inconsistent diagnostic definitions and variable comorbidity burdens. These inconsistencies limit cross-study integration. Prospective studies should pre-specify biologically informed endophenotypes (e.g., inflammatory, fibrotic, metabolic, vascular) and adopt standardized phenotyping protocols [16,17]. These may include modalities such as invasive or exercise hemodynamics, cardiac MRI T1/ECV mapping, and NP thresholds adjusted for BMI to enable downstream harmonization. Leveraging longitudinal phenotyping—including repeated measures of exercise capacity, congestion status, and circulating biomarkers—can also help distinguish transient states from stable endotypes, improving the interpretability of multi-omics signatures.

### 6.2. Batch Effects and Platform Drift

Technical variation between study sites or assay platforms can introduce systematic biases that mask true biological differences. To minimize these “batch effects,” investigators should standardize sample processing and analysis workflows, include shared reference samples for calibration, and apply statistical correction methods such as ComBat or similar batch-aware normalization models [126]. Transparent reporting of quality-control criteria, handling of missing data, and sensitivity analyses further strengthens reproducibility and comparability across studies [127]. As multi-omics expands across proteomic, metabolomic, and single-cell platforms, cross-platform drift becomes increasingly relevant; harmonizing metadata (e.g., instrument type, acquisition parameters, tissue source) and implementing joint normalization pipelines are essential for integrating datasets generated over multiple years or at multiple institutions.

### 6.3. Causality vs. Correlation

Most multi-omics associations describe correlation rather than true cause-and-effect relationships. To strengthen causal inference, genetic approaches such as Mendelian randomization or colocalization analysis can be used to test whether genetic variants linked to a molecular trait also predict disease outcomes [128]. Experimental perturbation—using CRISPR-based editing, RNA interference, or pharmacologic modulation in organoids or ex vivo tissue—can then validate whether altering a candidate gene or pathway modifies disease-relevant phenotypes or molecular programs [129,130]. Integration of causal models with temporal data—such as time-resolved proteomics or post-intervention transcriptomics—further helps distinguish upstream drivers from downstream consequences of HFpEF.

### 6.4. Cost and Scalability

Comprehensive multi-omics profiling across large cohorts remains costly and logistically demanding [131]. A practical solution is a two-stage design: deep multi-omics profiling in well-phenotyped HFpEF discovery cohorts to identify endotype-specific pathways, followed by validation in larger populations using targeted protein or metabolite panels. Collaborative biobanks, cross-institutional consortia, and emerging privacy-preserving analytic frameworks (e.g., federated learning) enable multi-omics studies to scale sample size and statistical power without requiring raw data transfer, as demonstrated by large machine learning-based analyses in UK Biobank [92]. Public–private partnerships and integration with health-system biorepositories (e.g., EHR-linked biobanks) can also reduce per-sample cost while enabling the recruitment of underrepresented demographic groups, which is essential for generalizable HFpEF biology.

### 6.5. Reproducibility and Data Sharing

Open and transparent data practices are essential for reproducibility. Following the FAIR principles—Findable, Accessible, Interoperable, and Reusable—investigators should deposit datasets in established public repositories such as PRoteomics IDentifications Exchange (PRIDE) or ProteomeXchange for proteomics, Gene Expression Omnibus (GEO) or ArrayExpress for transcriptomics, and MetaboLights for metabolomics. Sharing containerized analysis pipelines and version-controlled code ensures that published results can be fully reproduced and extended by other researchers. Clear documentation of analytical decisions (e.g., filtering thresholds, model hyperparameters, normalization methods) and inclusion of negative results or sensitivity analyses further increases transparency and reduces the “researcher degrees of freedom” that can compromise replicability.

## 7. Future Directions in Precision Cardiology

The next frontier in precision cardiology lies in uniting computational, biological, and clinical disciplines to transform how we diagnose, classify, and treat complex syndromes such as HFpEF. Emerging technologies and frameworks are converging to enable this shift, offering a path toward truly mechanism-guided cardiovascular medicine.

### 7.1. AI- and Machine Learning-Based Multi-Omics Integration

Artificial intelligence and machine learning approaches are increasingly essential for managing the scale and complexity of multi-omics data. By integrating heterogeneous datasets—genomics, transcriptomics, proteomics, metabolomics, and imaging—AI models can uncover hidden nonlinear relationships that traditional analyses miss. Deep learning architectures and graph neural networks are particularly well suited for identifying multi-layer molecular signatures that predict disease trajectory, drug response, or adverse outcomes. Importantly, explainable AI frameworks can translate these signatures into interpretable biological hypotheses, revealing causal drivers within the HFpEF network and accelerating therapeutic discovery.

### 7.2. Cross-Organ Multi-Omics and the Systems View of Heart Failure

HFpEF exemplifies a disorder rooted not only in myocardial dysfunction but also in multi-organ cross-talk. Future research will rely on cross-organ multi-omics profiling—integrating cardiac, renal, hepatic, adipose, and skeletal-muscle datasets—to map the bidirectional interactions that sustain disease. For example, the cardiorenal axis illustrates how renal venous congestion and accumulation of uremic toxins in HF/CKD promote systemic inflammation and adverse myocardial remodeling [59], while the cardiohepatic axis connects hepatic congestion and hypoxia—as well as HF-associated alterations in bile-acid metabolism—to downstream metabolic disturbances [132,133]. Systems-level integration of these datasets will illuminate how organ-specific perturbations propagate through shared molecular pathways, enabling targeted interventions that restore cross-organ homeostasis rather than focusing on a single organ.

### 7.3. Precision-Guided Clinical Trial Design

Future clinical trials in HFpEF must evolve from population-level inclusion criteria toward biologically enriched, mechanism-guided designs. Multi-omics and imaging-derived biomarkers can stratify patients into actionable endotypes—such as inflammatory, fibrotic, or metabolic HFpEF—and serve as both eligibility criteria and pharmacodynamic readouts. Adaptive and platform trial structures can further test multiple interventions in parallel, aligning treatment arms to molecular profiles. For instance, antifibrotic therapies could be preferentially tested in patients with elevated collagen biomarkers or T1/ECV on cardiac MRI, while metabolic modulators could target those with altered acylcarnitine or BCAA signatures. Similarly, therapies targeting NO-sGC-cGMP signaling (e.g., vericiguat) exemplify pathway-specific interventions that are mechanistically aligned with endothelial dysfunction-dominant HFpEF endotypes [134]. Such biomarker-guided enrichment increases the probability of detecting true therapeutic effects while reducing trial size and cost.

### 7.4. Collaborative Infrastructure and FAIR Data Ecosystems

Realizing precision cardiology at scale will require collaborative infrastructure that promotes data standardization, interoperability, and equitable access. Multi-center consortia integrating clinical phenotypes with multi-omics and imaging datasets—such as TOPMed, GTEx, and emerging HFpEF biobanks—are critical to achieving sufficient statistical power and external validation. Adherence to FAIR data principles will ensure transparency and reproducibility across platforms. Cloud-based federated learning and secure data-sharing frameworks can further enable cross-institutional analyses without transferring sensitive patient data, fostering a culture of open yet responsible collaboration.

Together, these advances will transform HFpEF research from descriptive clustering to predictive, mechanistic, and intervention-ready modeling, ultimately realizing the promise of precision cardiology—where the right therapy is delivered to the right patient, guided by molecular insight rather than clinical features alone.

## 8. Take-Home Messages

- HFpEF is not a single disease, but a heterogeneous syndrome composed of inflammatory, fibrotic, cardiometabolic, and hemodynamic/vascular endophenotypes.
- Traditional diagnostic tools (LVEF, natriuretic peptides, standard imaging) inadequately capture HFpEF biology and contribute to heterogeneous trial populations.
- Neutral or modest results of prior HFpEF trials largely reflect biological dilution rather than therapeutic inefficacy.
- Multi-omics approaches enable molecular stratification of HFpEF beyond clinical features, revealing mechanistically coherent endotypes.
- Integrative systems-biology frameworks linking genomics, transcriptomics, proteomics, and metabolomics can identify causal drivers, biomarkers, and drug targets.
- Network-based and genetic methods strengthen causal inference and prioritize actionable therapeutic targets.
- Biomarker-guided enrichment strategies offer a path toward mechanism-matched, precision clinical trials in HFpEF.
- Cross-organ and longitudinal multi-omics profiling is essential to capture HFpEF’s systemic nature.
- Successful implementation of precision cardiology will require standardized phenotyping, scalable omics pipelines, and FAIR data-sharing practices.

## 9. Conclusions

In conclusion, HFpEF represents both a clinical challenge and a unique proving ground for precision medicine. Its heterogeneity encapsulates the broader struggle of cardiovascular research—to move beyond symptom-based definitions toward biologically grounded classification. Advances in multi-omics integration, coupled with emerging AI and imaging analytics, are beginning to disentangle the molecular networks that drive inflammation, fibrosis, and metabolic dysregulation in HFpEF. These same frameworks can be applied across complex cardiovascular disorders, offering a blueprint for mechanism-guided diagnostics and targeted therapies. Achieving this transformation will depend on open data sharing, interdisciplinary collaboration, and the incorporation of molecular biomarkers into trial design—ensuring that precision cardiology becomes not an aspiration, but a clinical reality.

## Figures and Tables

**Table 1:** Major HFpEF Endophenotypes, Key Mechanisms, and Clinical Features.

Endophenotype	Core Clinical, Mechanistic, and Biomarker Features	Representative Therapeutic Approaches
Inflammatory	Obesity, diabetes, hypertension, CKD; cytokine-mediated endothelial activation; NO–cGMP–PKG impairment; oxidative stress; ↑ IL-6, TNF-α, CRP; inflammatory pathways on transcriptomics/proteomics	Anti-inflammatory approaches; SGLT2i; RAAS modulation; weight loss; CKD-directed therapies
Fibrotic/Increased Myocardial Stiffness/Remodeling	Elderly hypertensive women; LVH; TGF-β activation; collagen deposition; altered titin phosphorylation; ↑ PICP/PIIINP; galectin-3; ECM activation signatures; ↑ T1/ECV on CMR	Anti-fibrotics; RAAS/ARNI in selected patients; intensive BP control
Cardiometabolic/Obese	Obesity, IR, dyslipidemia, NAFLD; lipotoxicity; mitochondrial dysfunction; microvascular rarefaction; altered acylcarnitines/BCAA metabolism; low NP levels	SGLT2i; GLP-1RA; weight loss; metabolic modulation
Hemodynamic/Vascular	Older age; long-standing HTN; arterial stiffness; pulmonary hypertension; RV dysfunction; abnormal ventricular–vascular coupling; endothelin/vascular remodeling signatures	Therapies targeting vascular stiffness; pulmonary vasodilators in select phenotypes; exercise training

Symbol: ↑ indicates increased/elevated levels. Abbreviations: BCAA, branched-chain amino acids; BP, blood pressure; CKD, chronic kidney disease; CMR, cardiac magnetic resonance; CRP, C-reactive protein; cGMP, cyclic guanosine monophosphate; ECM, extracellular matrix; ECV, extracellular volume; HTN, hypertension; IL-6, interleukin-6; IR, insulin resistance; LVH, left ventricular hypertrophy; NAFLD, nonalcoholic fatty liver disease; NO, nitric oxide; NP, natriuretic peptides; PICP, procollagen type I C-terminal propeptide; PIIINP, procollagen type III N-terminal propeptide; PKG, protein kinase G; RAAS, renin–angiotensin–aldosterone system; RV, right ventricular; SGLT2i, sodium–glucose cotransporter-2 inhibitor(s); T1, native T1; TGF-β, transforming growth factor beta.

**Table 2:** Major HFpEF Clinical Trials, Key Findings, and Implications for Omics-Guided Trial Design.

Trial (Year) & Intervention	Key Findings and Heterogeneity Signals	Implications for Omics-Guided Trial Design
TOPCAT (2014) Spironolactone vs. placebo	Neutral primary endpoint. Benefit in Americas subgroup. Heterogeneity due to regional differences and NP-based vs. hospitalization entry; adherence issues; latent-class-defined subphenotypes with differential response.	Need biologically consistent inclusion criteria. Use omics to define inflammatory/fibrotic subphenotypes most likely to benefit.
PARAGON-HF (2019) Sacubitril–valsartan vs. valsartan	Narrow miss on primary composite endpoint. Signals of benefit in EF 45–57% and in women.	Rather than EF, future trials should stratify by vascular stiffness, fibrosis, and sex-specific pathways.
EMPEROR-Preserved (2021) Empagliflozin vs. placebo	Reduced HF hospitalizations; neutral CV mortality. Benefits especially in EF 41–49% (“HFmrEF”). Consistent across diabetes status.	Metabolic–renal pathways cut across EF. Omics can identify metabolic endotypes with strongest SGLT2i response.
DELIVER (2022) Dapagliflozin vs. placebo (EF > 40%, including improved EF)	Similar reduction in HF hospitalizations as EMPEROR. No major heterogeneity.	Broad EF inclusion is viable when mechanism is systemic. Future trials should apply omics to cluster metabolic vs. fibrotic vs. inflammatory responders.

**Abbreviations:** CV, cardiovascular; EF, ejection fraction; HF, heart failure; HFmrEF, heart failure with mildly reduced ejection fraction; HFpEF, heart failure with preserved ejection fraction; NP, natriuretic peptide(s); SGLT2i, sodium–glucose cotransporter-2 inhibitor(s). Trial acronyms: TOPCAT, Treatment of Preserved Cardiac Function Heart Failure with an Aldosterone Antagonist; PARAGON-HF, Prospective Comparison of Angiotensin Receptor–Neprilysin Inhibitor with Angiotensin Receptor Blocker Global Outcomes in Heart Failure with Preserved Ejection Fraction; EMPEROR-Preserved, Empagliflozin Outcome Trial in Patients with Chronic Heart Failure with Preserved Ejection Fraction; DELIVER, Dapagliflozin Evaluation to Improve the Lives of Patients with Preserved Ejection Fraction Heart Failure.

## Data Availability

No new data were created or analyzed in this study. Data sharing is not applicable to this article.

## Abbreviations

The following abbreviations are used in this manuscript:

ACEi	Angiotensin-Converting Enzyme Inhibitor
ARB	Angiotensin Receptor Blocker
ARNI	Angiotensin Receptor–Neprilysin Inhibitor
BCAA	Branched-Chain Amino Acids
NP	Natriuretic Peptide
BNP	B-type Natriuretic Peptide
cGMP	Cyclic Guanosine Monophosphate
CRISPR	Clustered Regularly Interspaced Short Palindromic Repeats
DELIVER	Dapagliflozin Evaluation to Improve the Lives of Patients with Preserved Ejection Fraction Heart Failure
ECV	Extracellular Volume
EMPEROR-Preserved	Empagliflozin Outcome Trial in HFpEF
eQTL	Expression Quantitative Trait Locus
FAIR	Findable, Accessible, Interoperable, and Reusable
HF	Heart Failure
HFmrEF	Heart Failure with Mildly Reduced Ejection Fraction
HFpEF	Heart Failure with Preserved Ejection Fraction
HFrEF	Heart Failure with Reduced Ejection Fraction
LVEF	Left Ventricular Ejection Fraction
MOFA	Multi-Omics Factor Analysis
MRI	Magnetic Resonance Imaging
MRA	Mineralocorticoid Receptor Antagonist
NO	Nitric Oxide
NT-proBNP	N-terminal pro–B-type Natriuretic Peptide
PARAGON-HF	Prospective Comparison of ARNI with ARB Global Outcomes in HF with Preserved Ejection Fraction
PICP	Procollagen Type I C-Peptide
PIIINP	Procollagen Type III N-Terminal Propeptide
PKG	Protein Kinase G
pQTL	Protein Quantitative Trait Locus
QTL	Quantitative Trait Locus
RNA-seq	RNA Sequencing
RV	Right Ventricle/Right Ventricular
SGLT2	Sodium–Glucose Cotransporter-2
smFISH	Single-Molecule Fluorescence In Situ Hybridization
TOPCAT	Treatment of Preserved Cardiac Function Heart Failure with an Aldosterone Antagonist
TWAS	Transcriptome-Wide Association Study
WGCNA	Weighted Gene Co-Expression Network Analysis
CKD	Chronic Kidney Disease
CMR	Cardiovascular Magnetic Resonance
RAAS	Renin–Angiotensin–Aldosterone System
NAFLD	Nonalcoholic Fatty Liver Disease
HTN	Hypertension
CCA	Canonical Correlation Analysis
PRIDE	PRoteomics IDentifications Exchange
GEO	Gene Expression Omnibus
